# Relapse Patterns in NMOSD: Evidence for Earlier Occurrence of Optic Neuritis and Possible Seasonal Variation

**DOI:** 10.3389/fneur.2020.00537

**Published:** 2020-06-16

**Authors:** Elham Khalilidehkordi, Laura Clarke, Simon Arnett, Wajih Bukhari, Sofia Jimenez Sanchez, Cullen O'Gorman, Jing Sun, Kerri M. Prain, Mark Woodhall, Roger Silvestrini, Christine S. Bundell, David Abernethy, Sandeep Bhuta, Stefan Blum, Mike Boggild, Karyn Boundy, Bruce J. Brew, Matthew Brown, Wallace Brownlee, Helmut Butzkueven, William M. Carroll, Celia Chen, Alan Coulthard, Russell C. Dale, Chandi Das, Marzena J. Fabis-Pedrini, David Fulcher, David Gillis, Simon Hawke, Robert Heard, Andrew P. D. Henderson, Saman Heshmat, Suzanne Hodgkinson, Trevor J. Kilpatrick, John King, Chris Kneebone, Andrew J. Kornberg, Jeannette Lechner-Scott, Ming-Wei Lin, Christopher Lynch, Richard A. L. Macdonell, Deborah F. Mason, Pamela A. McCombe, Jennifer Pereira, John D. Pollard, Sudarshini Ramanathan, Stephen W. Reddel, Cameron Shaw, Judith Spies, James Stankovich, Ian Sutton, Steve Vucic, Michael Walsh, Richard C. Wong, Eppie M. Yiu, Michael H. Barnett, Allan G. Kermode, Mark P. Marriott, John Parratt, Mark Slee, Bruce V. Taylor, Ernest Willoughby, Fabienne Brilot, Angela Vincent, Patrick Waters, Simon A. Broadley

**Affiliations:** ^1^Menzies Health Institute Queensland, Gold Coast Campus, Griffith University, Southport, QLD, Australia; ^2^Division of Immunology, HSQ Pathology Queensland Central Laboratory, Herston, QLD, Australia; ^3^Nuffield Department of Clinical Neurosciences, John Radcliffe Infirmary, University of Oxford, Oxford, United Kingdom; ^4^Department of Immunopathology, Westmead Hospital, Westmead, NSW, Australia; ^5^School of Pathology and Laboratory Medicine, University of Western Australia, Nedlands, WA, Australia; ^6^Department of Neurology, Wellington Hospital, Newtown, United Kingdom; ^7^Department of Neurology, Princess Alexandra Hospital, Woolloongabba, QLD, Australia; ^8^Department of Neurology, Townsville University Hospital, Douglas, QLD, Australia; ^9^Department of Neurology, Royal Adelaide Hospital, Adelaide, SA, Australia; ^10^Peter Duncan Neurosciences Unit, Centre for Applied Medical Research and Department of Neurology, St Vincent's Hospital, University of New South Wales, Darlinghurst, NSW, Australia; ^11^Institute of Health Biomedical Innovation, Translational Research Institute, Queensland University of Technology, Woolloongabba, QLD, Australia; ^12^Department of Neurology, Auckland City Hospital, Grafton, New Zealand; ^13^Department of Neuroscience, Central Clinical School, Monash University, Melbourne, VIC, Australia; ^14^Centre for Neuromuscular and Neurological Disorders, Perron Institute for Neurological and Translational Science, Queen Elizabeth II Medical Centre, University of Western Australia, Nedlands, WA, Australia; ^15^Flinders Medical Centre, Flinders University, Bedford Park, SA, Australia; ^16^School of Medicine, Royal Brisbane and Women's Hospital, University of Queensland, Herston, QLD, Australia; ^17^Westmead Clinical School, Westmead Hospital, University of Sydney, Westmead, NSW, Australia; ^18^Department of Neurology, Canberra Hospital, Garran, ACT, Australia; ^19^Sydney Medical School, Royal Prince Alfred Hospital, University of Sydney, Camperdown, NSW, Australia; ^20^Department of Neurology, Westmead Hospital, Westmead, NSW, Australia; ^21^South Western Sydney Medical School, Liverpool Hospital, University of New South Wales, Liverpool, NSW, Australia; ^22^Florey Institute of Neuroscience and Mental Health, University of Melbourne, Parkville, VIC, Australia; ^23^Department of Neurology, Royal Melbourne Hospital, Parkville, VIC, Australia; ^24^School of Paediatrics, Royal Children's Hospital, University of Melbourne, Parkville, VIC, Australia; ^25^Hunter Medical Research Institute, University of Newcastle, Callaghan, NSW, Australia; ^26^School of Medicine, University of Auckland, Grafton, New Zealand; ^27^Department of Neurology, Austin Health, Heidelberg, VIC, Australia; ^28^Department of Neurology, Christchurch Hospital, Christchurch, New Zealand; ^29^Centre for Clinical Research, Royal Brisbane and Women's Hospital, University of Queensland, Herston, QLD, Australia; ^30^Brain Autoimmunity Group, Institute for Neuroscience and Muscle Research, The Kids Research Institute at the Children's Hospital, Westmead, NSW, Australia; ^31^Brain and Mind Research Institute, University of Sydney, Camperdown, NSW, Australia; ^32^School of Medicine, Deakin University, Waurn Ponds, VIC, Australia; ^33^Menzies Institute for Medical Research, University of Tasmania, Hobart, TAS, Australia; ^34^Department of Neurology, St Vincent's Hospital, Darlinghurst, NSW, Australia

**Keywords:** neuromyelitis optica, multiple sclerosis, aquaporin, epidemiology, relapse, seasonality

## Abstract

Neuromyelitis optica spectrum disorders (NMOSD) and multiple sclerosis (MS) show overlap in their clinical features. We performed an analysis of relapses with the aim of determining differences between the two conditions. Cases of NMOSD and age- and sex-matched MS controls were collected from across Australia and New Zealand. Demographic and clinical information, including relapse histories, were recorded using a standard questionnaire. There were 75 cases of NMOSD and 101 MS controls. There were 328 relapses in the NMOSD cases and 375 in MS controls. Spinal cord and optic neuritis attacks were the most common relapses in both NMOSD and MS. Optic neuritis (*p* < 0.001) and area postrema relapses (*P* = 0.002) were more common in NMOSD and other brainstem attacks were more common in MS (*p* < 0.001). Prior to age 30 years, attacks of optic neuritis were more common in NMOSD than transverse myelitis. After 30 this pattern was reversed. Relapses in NMOSD were more likely to be treated with acute immunotherapies and were less likely to recover completely. Analysis by month of relapse in NMOSD showed a trend toward reduced risk of relapse in February to April compared to a peak in November to January (*P* = 0.065). Optic neuritis and transverse myelitis are the most common types of relapse in NMOSD and MS. Optic neuritis tends to occur more frequently in NMOSD prior to the age of 30, with transverse myelitis being more common thereafter. Relapses in NMOSD were more severe. A seasonal bias for relapses in spring-summer may exist in NMOSD.

## Introduction

Neuromyelitis optica spectrum disorders (NMOSD) have been recognized as having a distinct clinical and radiological phenotype which helps to differentiate these patients from those with multiple sclerosis (MS) (1). Early studies had indicated that the pathology of these two disorders was quite distinct, with NMOSD being more destructive (2). The identification of antibodies to the water channel aquaporin-4 (AQP4) in a significant proportion of patients with NMOSD (3) has greatly aided the diagnosis and treatment of this condition. The response to both acute relapse treatments and long-term preventive therapies are quite different for NMOSD and MS.

We have previously reported on the incidence and prevalence (4), AQP4 antibody assay findings (5) and clinical features (6) of a sizeable cohort of NMOSD cases meeting the 2015 International Panel for NMO Diagnosis (IPND) diagnostic criteria (1) collected from Australia and New Zealand. Here we analyze the specific details of relapse patterns, use of acute therapies and temporal patterns both in relation to the calendar year and across the lifespan of the disease. These data are compared with an age- and sex-matched cohort of MS cases collected from the same region with the aim of identifying distinct patterns of relapse that might further assist in the early identification of cases of NMOSD and provide information about potential trigger factors.

## Methods

### Case Ascertainment

This was a retrospective case-control study of NMOSD cases and MS controls. Cases of suspected NMOSD and MS were referred by a network of 23 clinical centres in Australia and New Zealand specializing in the assessment of patients with inflammatory diseases of the central nervous system in both adult and pediatric populations as previously described (4, 6). Cases of NMOSD were defined according to the 2015 IPND criteria (1). Testing for AQP4 antibodies was undertaken using either a tissue-based immunofluorescence technique or positivity on a least two cell-based assays (fixed, Euroimmun® or live, Oxford) as previously described (5). Testing for MOG antibodies was conducted using a live cell-based assay as previously described (5). Age- and sex-matched MS cases were identified from each centre with the diagnosis of MS being confirmed according to the 2010 McDonald criteria (7) with the added requirements of having no clinical features suspicious for NMOSD and being negative for AQP4 antibodies. Basic demographic and clinical features were recorded for all cases and controls as per a standardized data collection questionnaire as previously described (6). All participants provided written informed consent and the study was approved by the human research ethics committee of all participating institutions.

### Relapse Definitions

For relapses, data regarding the date of onset, symptoms experienced, presumed lesion location, treatment (intravenous steroids, plasma exchange, or intravenous gammaglobulin), maximal expanded disability status scale (EDSS), visual acuity, extent of recovery (full, partial or none), laterality (unilateral, bilateral or multicentric) was recorded for each relapse. Details of symptoms were provided by the participants and where available corroborated by reference to contemporaneous medical records and MR imaging findings. The precision for the date of onset was recorded as being either the day (date confirmed by medical records or patient diary reference), month (patient recollection or indirect medical records) or year (patient recollection). Lesion locations were based on symptomatology according to the following conventions. Motor, sensory, bladder, and pain symptoms in the limbs were attributed to a lesion of the spinal cord, unless there were additional brainstem or cerebral signs, or there was evidence of an active lesion elsewhere on MR imaging that could account for the symptoms in the absence of a relevant lesion in the spinal cord. Symptoms in the limbs with either ataxia, vestibular symptoms or cranial nerve signs were deemed to be a lesion of the brainstem/cerebellum. Hemi-motor or sensory symptoms were attributed to a lesion of the cerebral hemisphere where there was involvement of the face, cortical signs or a relevant hemispheric lesion. Blurring of vision in one or both eyes was deemed to be due to a lesion of the optic nerve, chiasm or tracts, unless there were additional brainstem signs. If symptoms could not be attributed to a single lesion site or if there was evidence of multiple active lesions on MR imaging, then lesions were deemed to be multifocal and assigned to the smallest number of regions required to explain all the symptoms. Episodes of hiccoughs, nausea and vomiting with a lesion of the area postrema evident on MR imaging were counted as area postrema relapses. Encephalitic presentations were defined as focal hemispheric symptoms or a focal hemispheric lesion associated with seizures, headache or clouding of consciousness. Classical Devic presentations were defined as the simultaneous or sequential onset (within 3 months) of optic neuritis and transverse myelitis (8).

### Statistical Analysis

Frequencies are expressed as n/N (%) and continuous data are presented as median (range) if not normally distributed or mean (SD) if normally distributed. Comparisons between NMOSD and MS have been made using appropriate parametric or non-parametric tests. For categorical variables, Fisher's exact test was used when the number of patients in any cell was less than five. No correction for multiple testing was undertaken. These statistical tests were performed using Statistical Package for Social Science (SPSS®) v25 (IBM® Chicago, US). Auto regressive integrated moving average time series method was used to analyze the effect of month and seasons in the time series to predict the occurrence of relapse in MS. Relapse counts were analyzed by month using a Poisson regression model with the median month of relapse used as the reference, as has been used previously in MS (9). These analyses were performed using the STATA® statistical package v14 (StataCorp® College Station, Texas, US).

## Results

### NMOSD Cases and MS Controls

**Figure 1:**
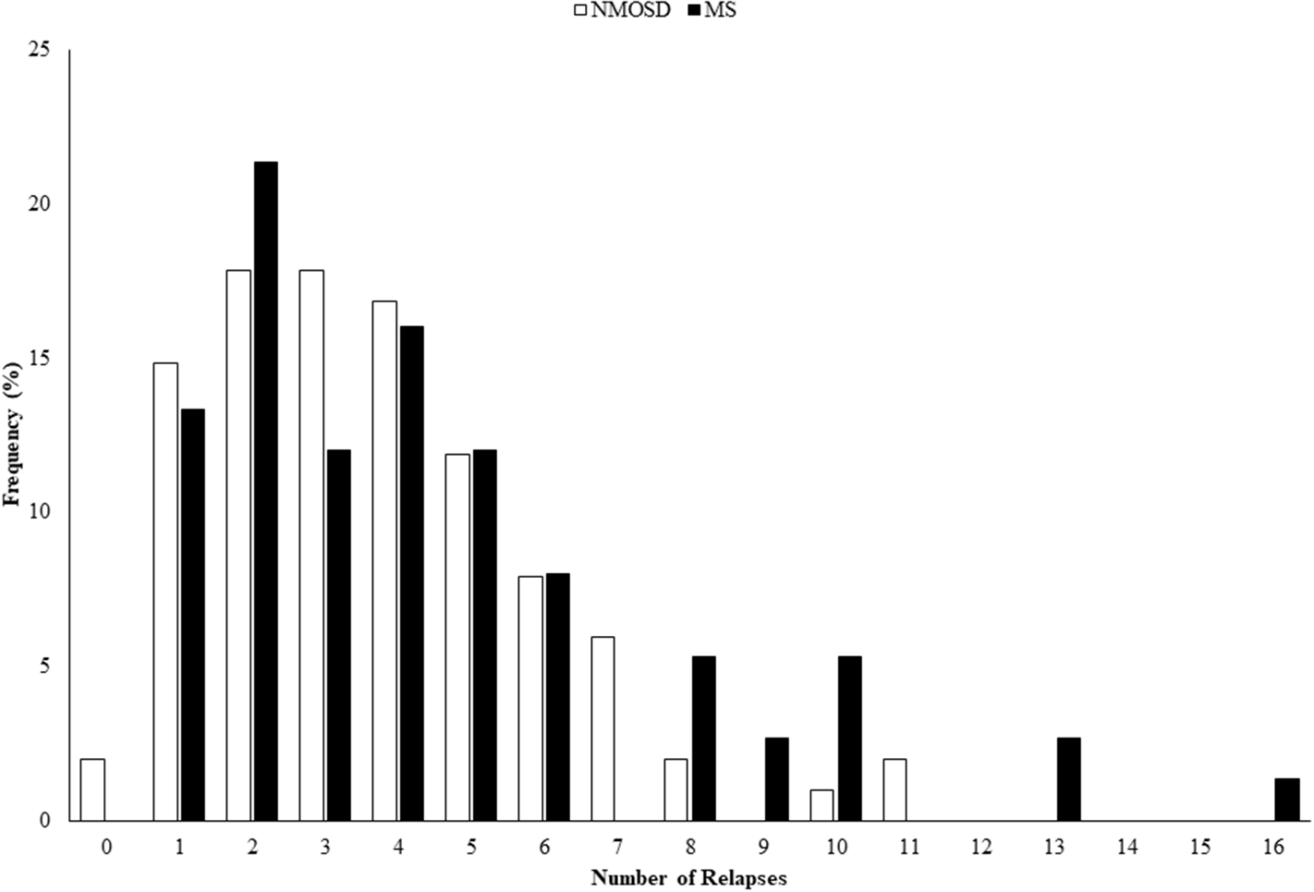
Histogram showing distribution of numbers of relapses seen in NMOSD and MS. NMOSD, neuromyelitis optica spectrum disorder; MS, multiple sclerosis.

There were 75 cases of NMOSD with full clinical data that met the 2015 IPND criteria (1), of which 68 (91%) were positive for AQP4 antibodies. There were 101 controls with MS who were all negative for AQP4 antibodies and met the 2010 McDonald criteria (7). Testing for MOG antibodies was conducted on 42/75 (56%) of NMOSD cases, including all of the seronegative cases and 52/101 (51%) of MS controls, and all were negative (5). The demographic and clinical features of the NMOSD cases and MS controls have been previously reported (6) and show that they were well matched for age and sex, but differ in a number of predictable clinical features as summarized in Table 1. Age of onset in MS cases was younger and consequently disease duration was longer. Despite this the number of relapses seen in NMOSD was greater, although not significantly, and the annualized relapse rate was approximately double that of MS controls (*p* < 0.001). The distribution of numbers of relapses in the two groups is illustrated in Figure 1. The level of disability at last review was greater in NMOSD compared to MS (median EDSS 4.0 vs. 2.0; *p* < 0.001). Secondary progressive disease was only seen in two cases of NMOSD and primary progressive NMOSD was not seen. The proportion of cases with monophasic disease was similar for NMOSD and MS although the extent of follow up for the MS cases was greater. The proportion of NMOSD cases experiencing a classical Devic presentation showed a trend toward being higher than in MS and these presentations were more likely to involve bilateral optic neuritis or be sequential in NMOSD, but were more commonly recurrent in MS. However, none of these differences were statistically significant due to the small numbers.

**Table 1:** Comparison of clinical features of NMOSD and MS.

**Clinical feature**	**NMOSD**	**MS**	***p*-value**
*N*	75	101	
Age (Years)–median (range)	47 (19–85)	46 (16–73)	ns
Gender (Female)–*n*/*N* (%)	68/75 (91)	86/101 (85)	ns
Age at Onset (Years)–median (range)	40 (13–85)	32 (6–59)	0.001
Disease Duration (Years)–median	4.1 (0.1–43.1)	12.3 (0.5–43.3)	<0.001
(range)			
Relapses–median (range)	4 (1–16)	3 (0–11)	ns
Annualized relapse rate–median	0.77 (0.13–3.33)	0.33 (0.06–3.78)	<0.001
(range)			
EDSS–median (range)	4 (0–9)	2 (0–9)	<0.001
Clinical Course–*n* (%)			ns
Monophasic (CIS)	10 (13)	12 (12)	
Relapsing remitting	63 (84)	73 (72)	
Secondary progressive	2 (3)	13 (13)	
Primary progressive	0 (0)	3 (3)	
Classical Devic presentation–*n* (%)	12 (16)	9 (9)	ns
With bilateral optic neuritis	4/12 (33)	2/9 (22)	ns
Sequential (≤3 months)	6/12 (50)	1/9 (11)	ns
Recurrent	2/12 (17)	3/9 (33)	
Initial MR brain imaging	12/70 (17)	3/100 (3)	0.001
normal–*n*/*N* (%)			
LESCL on MR spine	48/71 (68)	1/89 (1)	<0.001
imaging–*n*/*N* (%)			

*NMOSD, neuromyelitis optica; MS, multiple sclerosis; LESCL, longitudinally extensive spinal cord lesion; CIS, clinically isolated syndrome; SD, standard deviation; EDSS, expanded disability status scale; ns, non-significant*.

### Types of Relapse

The frequency of different relapse types and lesion locations is summarized in Table 2. The proportions of relapse locations at first relapse and for all relapses were similar within the two cohorts (Table 2). However, there was a difference between NMOSD cases and MS controls in the frequency of optic neuritis (*p* < 0.001) and brainstem lesions (*p* < 0.001). Optic neuritis and area postrema lesions were more common in NMOSD and other brainstem lesions were more common in MS. Cerebral syndromes were rare in NMOSD and there was a trend toward these being more common in MS, but the overall numbers were lower, and this difference was not significant. There was only one encephalitis presentation seen in NMOSD. Area postrema syndromes were more common as a first relapse (9%) compared to all relapses (3%). There were no cases of NMOSD that presented with hypothermia, drowsiness or syndrome of inappropriate anti-diuretic hormone syndrome. When analyzed by sex and serostatus there were no significant differences in the pattern of relapse in NMOSD (data not shown).

**Table 2:** Frequency of relapse locations in NMOSD and MS.

**Relapse syndrome**	**First relapse**			**All relapses**		
	**NMOSD**	**MS**	***p*-value**	**NMOSD**	**MS**	***p*-value**
*n*	75	101		329	375	
Transverse myelitis	33 (44)	51 (50)	ns	159 (48)	165 (44)	ns
Optic neuritis	29 (38)	12 (12)	<0.001	131 (40)	62 (16)	<0.001
Area postrema syndrome	7 (9)	0 (0)	0.009	11 (3)	0 (0)	0.002
Other brainstem syndrome	3 (4)	25 (25)	<0.001	16 (5)	90 (24)	<0.001
Optic neuritis and transverse myelitis	2 (2)	3 (3)	ns	7 (2)	14 (4)	ns
Cerebral syndrome	0 (0)	5 (5)	ns	2 (1)	15 (4)	ns
Optic neuritis and brainstem syndrome	0 (0)	2 (2)	ns	2 (1)	9 (2)	ns
Brainstem syndrome and transverse myelitis	1 (1)	0 (0)	ns	1 (0.3)	0 (0)	ns

*NMOSD, neuromyelitis optica; MS, multiple sclerosis; ns, non-significant*.

**Figure 2:**
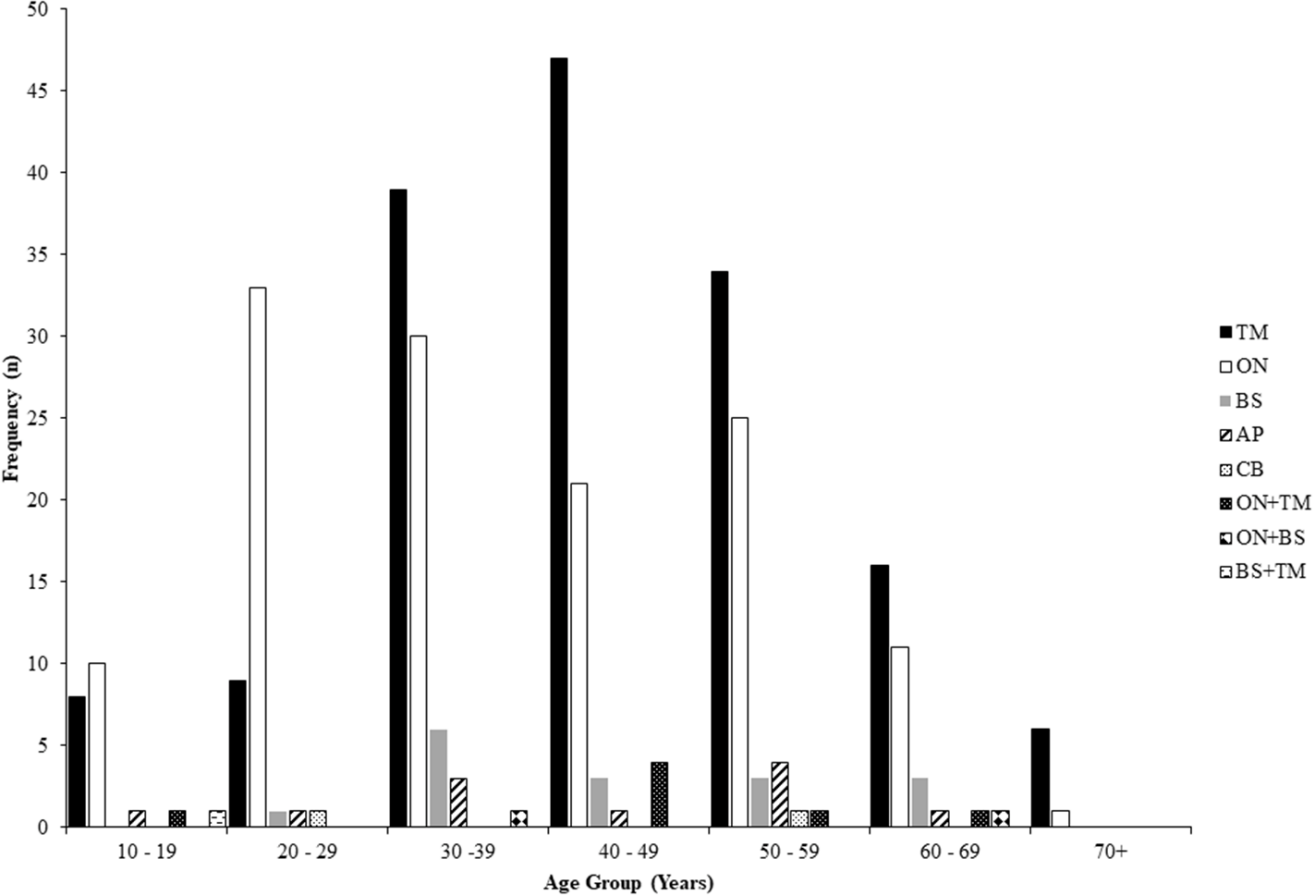
Frequency of relapse lesion locations according to age at the time of relapse in NMOSD. NMOSD, neuromyelitis optica spectrum disorder; TM, transverse myelitis; ON, optic neuritis; BS, brainstem/cerebellar; AP, area postrema; CB, cerebral.

The frequency of lesion location in NMOSD according to age at the time of relapse for all relapses is shown in Figure 2 and indicates that episodes of optic neuritis predominate at a younger age with a peak age at 20–29 years, whilst attacks of transverse myelitis predominate later with a peak incidence at 40–49 years. Relapses of all types were seen across a broad range of ages (10–69 years).

### Relapse Features, Treatment and Outcomes

The principal features, treatment and outcomes for all relapses in NMOSD and MS are given in Table 3. The time between relapses was shorter in NMOSD (10.6 months) compared with MS (18.0 months). There was no difference in the proportion of optic neuritis attacks that were bilateral in NMOSD and MS, but the absolute frequency was higher in NMOSD (21 vs. 8). Spinal cord relapses were more commonly partial in MS. Relapse duration and maximal disability level were greater in NMOSD. NMOSD cases were more likely to be treated with high dose intravenous or oral steroids, plasma exchange and intravenous immunoglobulin. Complete recovery from a relapse was more common (*p* < 0.001) in MS (56%) than NMOSD (29%).

**Table 3:** Comparison of relapse features, treatment and outcomes in NMOSD and MS.

**Relapse feature**	**NMOSD**	**MS**	***p*-value**
*N*	328	375	
Time between relapses	10.6 (0.3–336.0)	18.0 (0.5–408.4)	<0.001
(months)–median (range)			
Bilateral optic neuritis–*n*/*N* (%)	21/116 (18)	8/55 (15)	ns
Partial cord syndrome– *n*/*N* (%)	55/134 (41)	80/148 (54)	0.03
Relapse duration (days)–mean* (range)	68 (2–666)	50 (1—365)	<0.001
Maximal EDSS–median (range)	4 (1–10)	3 (1–8)	<0.001
Treated with IVMP–*n* (%)	193 (59)	149 (40)	<0.0001
Treated with PLEX–*n* (%)	41 (13)	0 (0)	<0.0001
Treated with IVIg–*n* (%)	20 (6)	2 (1)	<0.0001
Outcome– *n*/*N* (%)			<0.0001
Complete recovery	78/271 (29)	165/295 (56)	
Partial recovery	170/271 (63)	109/295 (37)	
No improvement	23/271 (8)	21/295 (7)	

* *Mean is given in place of median which was 30 days (1 month) for both NMOSD and MS*.

*NMOSD, neuromyelitis optica spectrum disorder; MS, multiple sclerosis; EDSS, expanded disability status scale; IVMP, intravenous methylprednisolone or very high dose oral steroids; PLEX, plasma exchange; IVIg, intravenous immunoglobulin; ns, non-significant*.

### Seasonal Variation in Relapses

**Figure 3:**
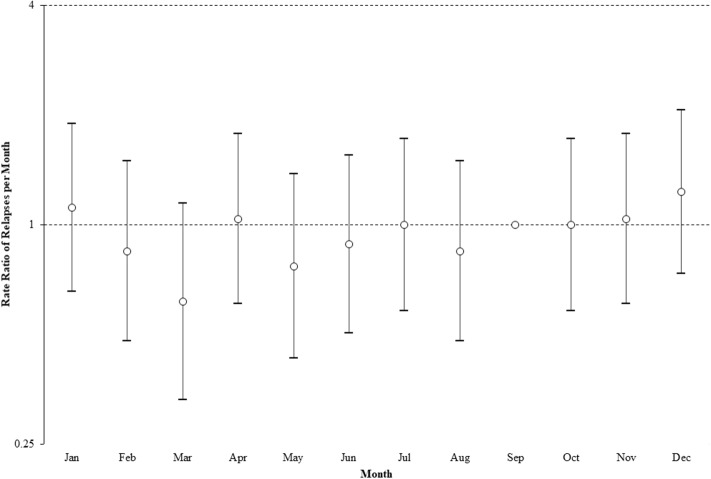
Rate ratio of relapses per month, using median of 29 relapses per month (September). Error bars show 95% confidence intervals (Poisson regression analysis). Y-axis plotted on logarithmic scale.

The seasonal pattern of relapses in NMOSD is shown in Figure 3. The auto regressive integrated moving average analysis indicated a marginal significance of month on number of relapses per month with coefficient = 0.531 C95% CI − 0.678 – 1.13, *P* = 0.082) and adjusted coefficient = 3.677 (95% CI 2.034–5.320, *P* < 0.001). Poisson regression analysis indicated that no individual month significantly deviated from the median (Figure 3). Analysis of 3-month époques indicated a trend toward fewer relapses in February to April compared to November to January (*P* = 0.065). This corresponds to a potential peak risk of relapse in mid-spring and summer in the Southern Hemisphere and is similar to the pattern seen in MS for this part of the world (10) which has been attributed to a 1–2 month lag in relapses after the nadir of vitamin D levels (September in the Southern Hemisphere) (10).

## Discussion

The present data indicate that the commonest relapse types seen in NMOSD are transverse myelitis and optic neuritis and that optic neuritis attacks, particularly as first attacks, are more common in NMOSD than MS. Area postrema presentations were exclusively seen in NMOSD and accounted for 9% of first relapses and 3% of all relapses. Attacks of optic neuritis were seen more frequently at a younger age in NMOSD with episodes of transverse myelitis occurring more frequently later. Relapse frequency, duration and severity, together with the requirement for acute immunotherapies, were all greater in NMOSD than MS. In the situation where the diagnosis of NMOSD had been established there would be a potential bias toward the use of acute immunotherapies.

As with previous studies the most frequent form of relapse in NMOSD was a lesion of the spinal cord and the frequency observed in the present study (48%) falls in the middle of previous observations (36–63%) (11–15). The frequencies of other relapse types were similar to these prior studies. As with previous studies relapses with encephalitic or other cerebral features were uncommon in NMOSD. Area postrema lesions as an initial presenting feature was seen in (9%) which was similar to prior studies (15). We found that area postrema relapses were more common at first presentation than with subsequent relapses. This finding is contrary to a recent larger study of several international cohorts (16). However, we note the definition for area postrema syndrome used in that study was broader than the definition used in the present study. No relapses involving hypothermia (17) or syndrome of inappropriate anti-diuretic hormone syndrome (18) were seen in our cohort.

Despite being an inclusion criterion for suspected NMOSD in our original clinical survey, there were eight optic neuritis attacks in our MS cohort that were bilateral. These were historical attacks and the lesion location was based on symptomatology which can be prone to error. For example, bilateral visual blurring can arise as a result of mild diplopia from a brainstem lesion or a homonymous field deficit due to a cerebral lesion. These cases otherwise had features typical for MS and were therefore not reclassified. Classical Devic presentations with either simultaneous or sequential optic neuritis and transverse myelitis were only marginally more common in NMOSD than MS and this was not a significant difference. Classical Devic presentations were seen in 16% of NMOSD cases. The exclusion from the MS controls of cases with features suspicious for NMOSD could potentially introduce a bias in the relapse features reported here. However, we would note that the number of cases referred with NMOSD-like features that did not meet 2015 IPND criteria was similar to the number of confirmed NMOSD cases in our original survey (6), thus representing no more than 1% of all MS cases. This is unlikely to introduce any significant bias. Recall bias is always a potential issue with retrospectively collected relapse data. However, the methods used in this study were identical for the NMOSD cases and MS controls.

The frequency with which high dose steroids were administered for attacks of NMOSD (58%) was higher than in MS and was similar to previous studies (65–84%) (12, 13). The frequency of complete recovery was lower in NMOSD than MS and was in a range similar to that observed previously (13).

Two novel findings in the present study are the observation that attacks of optic neuritis predominate in younger patients with NMOSD whilst transverse myelitis is more common later in life and that there is a seasonal variation in the frequency of attacks. An earlier study has noted the predominance of optic neuritis in first presentations prior to the age of 30 years, with transverse myelitis being more common above 30 (19). We are not aware of prior data looking at seasonal variability of relapses in NMOSD. A trend toward fewer relapses from February to April compared to a peak from November to January is similar to the pattern seen in MS both in the Northern and Southern Hemisphere (9, 10). This finding is somewhat surprising considering the absence of a latitudinal gradient seen in two national studies of NMOSD prevalence (4, 20). This suggests that relative vitamin D deficiency or decreased ultraviolet B radiation exposure are not significant factors in the risk of developing NMOSD but may be factors influencing the likelihood of relapses. These findings require confirmation in further studies.

In conclusion, we have confirmed the findings of prior studies with regard to the pattern of relapses and clinical features seen in NMOSD. We have shown that this pattern differs significantly from MS in a number of areas. There was no difference in the frequency of classical Devic presentations between NMOSD and MS, but there was a trend toward sequential and bilateral optic neuritis Devic's presentations being more common in NMOSD. The finding of optic neuritis attacks occurring more commonly at a younger age is interesting and as with the sequential optic nerve involvement with later spinal cord disease seen in classical Devic's syndrome suggests a specific vulnerability of the optic nerve early in the disease course. The increased risk of NMOSD relapse during the spring-summer suggests a seasonally dependent environmental risk factor influencing the timing of relapses in NMOSD.

## Data Availability Statement

The datasets generated for this study are available on request to the corresponding author.

## Ethics Statement

This study was reviewed and approved by Griffith University Human Research Ethics Committee (MED2009/646) and had local Governance Approval at each participating site. All participants gave written informed consent.

## Author Contributions

DA, MHB, SBh, SBl, MBo, KB, BB, SAB, MBr, WBr, HB, WC, CC, AC, RD, CD, DG, SHa, RH, AH, SHo, AGK, TJK, JK, CK, JL-S, CL, RM, MM, DM, PM, CO'G, JPa, JPe, JDP, KP, SWR, CS, MS, JSp, JSu, IS, BT, AV, SV, MWa, PW, EW, and RCW conceived and designed the study. EK, SAB, WBu, CB, LC, JSu, MF-P, DG, SHe, SJ, M-WL, KP, RS, JSt, BT, PW, MWo, and EY conducted the analyses. EK, WBu, and LC prepared the initial draft. AC, BB, FB, HB, RD, MF-P, SHa, SHo, JK, AJK, JL-S, PM, RM, SWR, JSi, BT, AV, SV, and PW contributed to revisions. All authors approved the final draft.

## Conflict of Interest

MHB has received honoraria for participation in advisory boards and travel sponsorship from Novartis, BioCSL, Genzyme and Biogen Idec. MBo has received travel sponsorship and honoraria from Sanofi-Genzyme, Teva, Novartis, Biogen Idec and Roche. BB has received honoraria as a board member for GlaxoSmithKline, Biogen Idec, ViiV Healthcare and Merck Serono, has received speaker honoraria from ViiV Healthcare, Boehringer Ingelheim, Abbott, Abbvie, and Biogen Idec; has received travel sponsorship from Abbott and Viiv Healthcare, and has received research support funding from EI Lilly, GlaxoSmithKline, ViiV Healthcare and Merck Serono. SAB has received honoraria for attendance at advisory boards and travel sponsorship from Bayer-Scherring, Biogen-Idec, Merck-Serono, Novartis, and Sanofi-Genzyme, has received speakers honoraria from Biogen-Idec and Genzyme, is an investigator in clinical trials sponsored by Biogen Idec, Novartis and Genzyme, and was the recipient of an unencumbered research grant from Biogen-Idec. HB has received honoraria for serving on scientific advisory boards for Biogen Idec, Novartis and Sanofi-Genzyme, has received conference travel sponsorship from Novartis and Biogen Idec, has received honoraria for speaking and acting as Chair at educational events organized by Novartis, Biogen Idec, Medscape and Merck Serono, serves on steering committees for trials conducted by Biogen Idec and Novartis, is chair (honorary) of the MSBase Foundation, which has received research support from Merck Serono, Novartis, Biogen Idec, Genzyme Sanofi and CSL Biopharma and has received research support form Merck Serono. WMC has been the recipient of travel sponsorship from, and provided advice to, Bayer Schering Pharma, Biogen-Idec, Novartis, Genzyme, Sanofi-Aventis, BioCSL, and Merck-Serono. RD has received research funding from the National Health and Medical Research Council, MS Research Australia, Star Scientific Foundation, Pfizer Neuroscience, Tourette Syndrome Association, University of Sydney, and the Petre Foundation and has received honoraria from Biogen-Idec and Bristol-Myers Squibb as an invited speaker MF-P has received travel sponsorship from Biogen Australia and New Zealand and Merck. RH has received honoraria, educational support and clinic funding from Novartis, Biogen Idec, Genzyme and BioCSL. AGK has received scientific consulting fees and/or lecture honoraria from Bayer, BioCSL, Biogen-Idec, Genzyme, Merck, Novartis, Sanofi-Aventis, and Teva. TK has received travel sponsorship from Novartis, BioCSL, Novartis, Merck Serono and Biogen Idec, has received speaker honoraria from Biogen Idec, BioCSL, Merck Serono, Teva, Genzyme and Novartis, has received research support from Biogen Idec, Genzyme, GlaxoSmithKline, Bayer-Schering and Merck Serono, and has received scientific consulting fees from GlaxoSmithKline China, Biogen-Idec and Novartis. JK has received remuneration for advisory board activities and presentations from Bayer Healthcare, Biogen Idec, BioCSL, Genzyme and Novartis. CK has received travel support, honoraria and advisory board payments from Biogen Idec, Bayer, Genzyme, Novartis and Serono. JL-S has received unencumbered funding as well as honoraria for presentations and membership on advisory boards from Sanofi Aventis, Biogen Idec, Bayer Health Care, CSL, Genzyme, Merck Serono, Novartis Australia and Teva. RM has received honoraria for attendance at advisory boards and travel sponsorship from Bayer-Scherring, Biogen-Idec, CSL, Merck-Serono, Novartis, and Sanofi-Genzyme. MM has received travel sponsorship, honoraria, trial payments, research and clinical support from Bayer Schering, Biogen Idec, BioCSL, Genzyme, Novartis and Sanofi Aventis Genzyme. DM has received honoraria for attendance at advisory boards from Biogen-Idec and Novartis, and travel sponsorship from Bayer-Scherring, Biogen-Idec, and Sanofi-Genzyme. PM has received honoraria or travel sponsorship from Novatis, Sanofi-Avnetis and Biogen Idec. JPa has received travel sponsorship, honoraria for presentations and membership on advisory boards from Biogen Idec and Novartis and Sanofi Aventis. JDP has received honoraria for seminars or advisory boards from Teva, Biogen, Sanofi-Genzyme, Novartis, Merck, Bayer and research grants or fellowships from Merck, Novartis, Bayer, Biogen, Sanofi-Genzyme and Teva SWR has received travel sponsorship, honoraria, trial payments, research and clinical support from Aspreva, Baxter, Bayer Schering, Biogen Idec, BioCSL, Genzyme, Novartis, Sanofi Aventis Genzyme and Servier, and is a director of Medical Safety Systems Pty Ltd. CS has received travel sponsorship from Biogen Idec, Novartis and Bayer-Schering. IS has received remuneration for Advisory Board activities from Biogen, CSL, and Bayer Schering and educational activities with Biogen, CSL and travel sponsorship from Biogen, Novartis and Bayer Schering. MS has received research support from Novartis, Biogen Idec and BioCSL. JSp has received honoraria for lectures and participation in advisory boards, and travel sponsorship from Novartis, BioCSL, Genzyme and Biogen Idec. BT has received travel sponsorship and honoraria for participation in advisory boards from Bayer Schering, Merck, Novartis and Roche. AV and the University of Oxford hold patents and receive royalties for antibody testing. PW and the University of Oxford hold patents for antibody assays and have received royalties, has received speaker honoraria from Biogen Idec and Euroimmun AG, and travel grants from the Guthy-Jackson Charitable Foundation. EW has received honoraria for participation in advisory boards from Biogen-Idec and Novartis, travel sponsorship from Biogen-Idec, Bayer-Schering and Teva and is an investigator in clinical trials funded by Biogen-Idec and Teva. The remaining authors declare that the research was conducted in the absence of any commercial or financial relationships that could be construed as a potential conflict of interest.
